# Tumor-derived granzyme B-expressing neutrophils acquire antitumor potential after lipid A treatment

**DOI:** 10.18632/oncotarget.25342

**Published:** 2018-06-19

**Authors:** Amandine Martin, Cédric Seignez, Cindy Racoeur, Nicolas Isambert, Nesrine Mabrouk, Alessandra Scagliarini, Sylvie Reveneau, Laurent Arnould, Ali Bettaieb, Jean-François Jeannin, Catherine Paul

**Affiliations:** ^1^ Laboratoire d'Immunologie et Immunothérapie des Cancers, EPHE, PSL Research University, 75000 Paris, France; ^2^ LIIC, EA7269, Université de Bourgogne Franche Comté, 21000 Dijon, France; ^3^ Centre Georges-François Leclerc, Dijon, F-21000, France

**Keywords:** tumor-associated neutrophils, granzyme B, colon cancer, immune response, lipid A

## Abstract

Neutrophils are known to possess both pro- and anti-tumor properties, a feature that could be related to the diversity and plasticity of these cells. Here we explored the hypothesis that under an appropriate environment and stimuli, neutrophils could induce an effective response against tumor cells. In a rat and mouse models, we show that a substantial amount of colon tumor associated-neutrophils (TAN) expressed the cytolytic enzyme granzyme B, which is absent in spleen or blood circulating neutrophils. This TAN population was also found into tumors of patients with colon cancer. Tumor neutrophil infiltration was correlated with an increase of chemokines known to attract neutrophils in both rat models and patients. These cells were involved in a Lipid A analog-mediated colon tumor regression. Mechanistically, treating the rats with the Lipid A analog triggered granzyme B release from neutrophils in tumor cell vicinity, which was correlated to tumor regression. Alteration of granzyme B function in tumor cells decreased the cytotoxic effect of Lipid A in rat and mouse models. Granzyme B expression in neutrophils could be induced by the lipid A analog but also by some of the cytokines that were detected in the tumor microenvironment. These results identify a subpopulation of neutrophils expressing granzyme B that can act as a key player of lipid A-mediated colon cancer regression in rat and mouse models and the molecular mechanisms involved may provide novel approaches for human therapeutic intervention.

## INTRODUCTION

A well-known example of cancer immunotherapy in use for more than 30 years is the Bacillus Calmette-Guérin (BCG) therapy of non-invasive bladder carcinomas. This is the first most effective immunotherapy for solid tumors [1]. The molecular mechanisms involved are likely dependent on the binding of BCG, an avirulent strain of *Mycobacterium bovis*, to the Toll-like receptors (TLR) 4 [2]. This TLR plays a critical role in the activation of the immune system by stimulating antigen uptake and presentation, maturation of dendritic cells (DC), differentiation of helper T cells, and inhibition of regulatory T cells [3].

TLR4 is the classic receptor of lipopolysaccharides (LPS) and lipid (A) [4], the lipid part of LPS to which the anti-tumor activity of LPS was attributed [5]. However, LPS and natural forms of lipid A are too toxic to be used in clinic. Lipid A analogs have been developed and some of them, like OM-174 (LipA), are well tolerated by patients [6] and effective against tumors in animal models. LipA is a triacyl diglucosamine diphosphate acting through TLR4 to induce total regression or growth inhibition of macroscopic tumors in rat and mouse models [7–11].

Immunotherapy results in tumor cell death. Granzymes, known to induce apoptosis, are the main mediators of cytotoxicity induced by cytotoxic T lymphocytes and natural killer (NK) cells [12, 13]. Over the last decade, there were some controversy about granzymes A and B expression in other cell types like neutrophils. Some reports show that granzymes are constitutively expressed in human blood neutrophils [14, 15] or transformed neutrophils in culture [16], while others indicate that granzymes are not at all expressed in neutrophils [17, 18]. The same controversy was extended to granzyme expression in mouse neutrophils [19]. Granzyme B (GZMB) expression in tumor neutrophils has not been studied. Even though neutrophils are known to support tumor growth by producing angiogenic factors like matrix metalloproteinase 9 and vascular endothelial growth factor [20], recent data demonstrate that the microenvironment can alter neutrophil activity. For example, blocking transforming growth factor beta (TGF-β1) induced the switch of neutrophils from a pro-tumorigenic (N2) to an anti-tumorigenic (N1) state [21].

As neutrophils were implicated in the mechanism of action of BCG immunotherapy [22–24], we sought to elucidate their role in LipA immunotherapy in rat and mouse models of colon cancer and we checked that human neutrophil exhibited the same properties.

## RESULTS

### The antitumor effect of the LipA treatment is correlated with the infiltration of tumors by neutrophils

**Figure 1:**
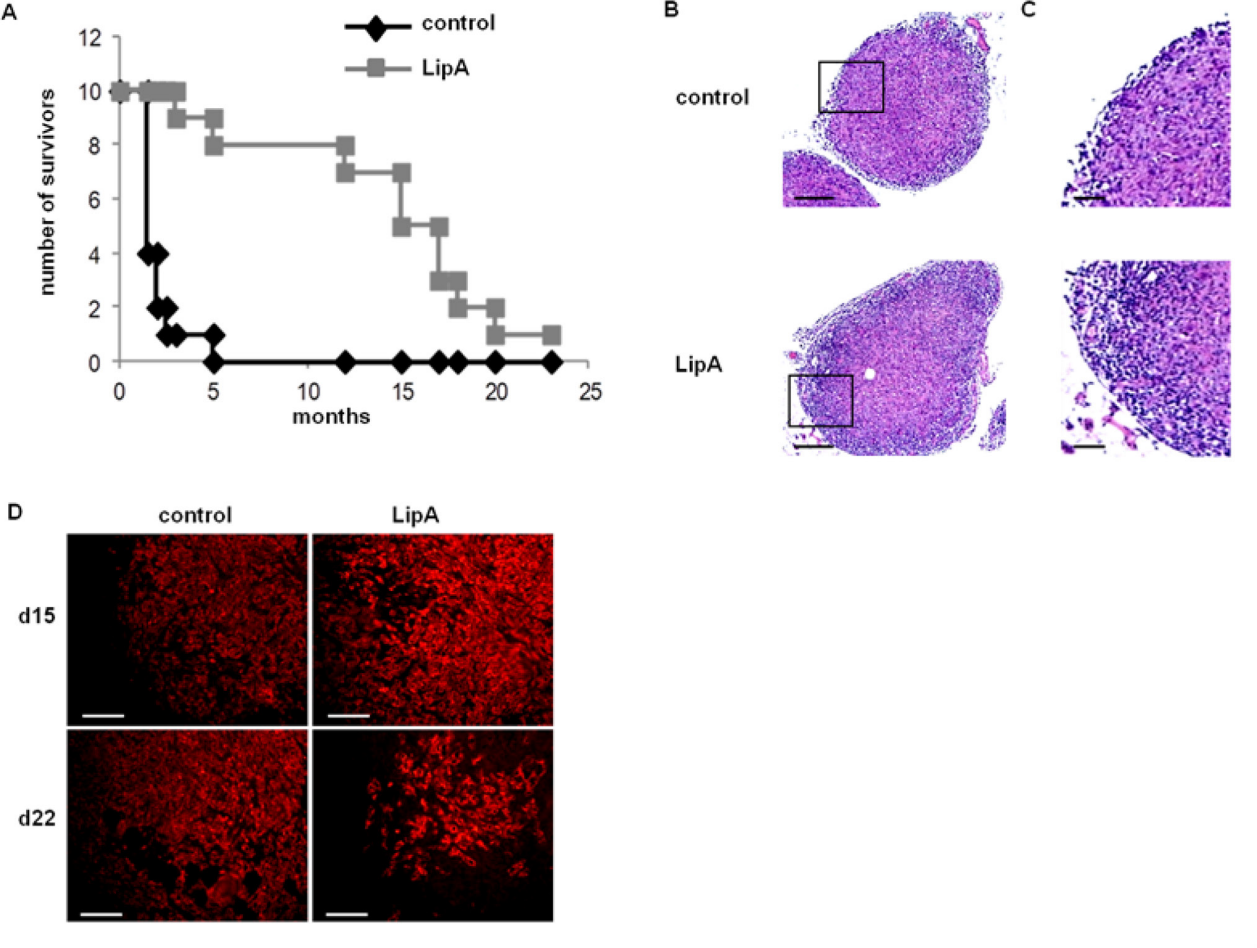
LipA cured tumor-bearing rats (**A**) The lifespan of tumor-bearing rats was improved by LipA treatment. Fourteen days after i.p. injection of PROb cells, rats were treated i.v. with LipA (grey) or physiological solution (control, black) 3 times per week for 5 weeks. Results are representative of at least 3 independent experiments with 10 animals per group. (**B**) Hematoxylin-eosin staining of tumors from LipA treated or control rats (scale bars = 200 µm). (**C**) Enlargement of regions outlined in figure B (scale bars = 50 µm). The tumor core and surrounding ring of cells can be distinguished. (**D**) Tumor regression was analyzed by immunostaining of tumor cells (anti-cytokeratin Ab, red), one day after the first (day 15) and the fourth injection (day 22) of LipA or physiological solution (scale bars = 50 µm). Micrographs are representative of at least 3 independent experiments, 4 animals per group.

**Figure 2:**
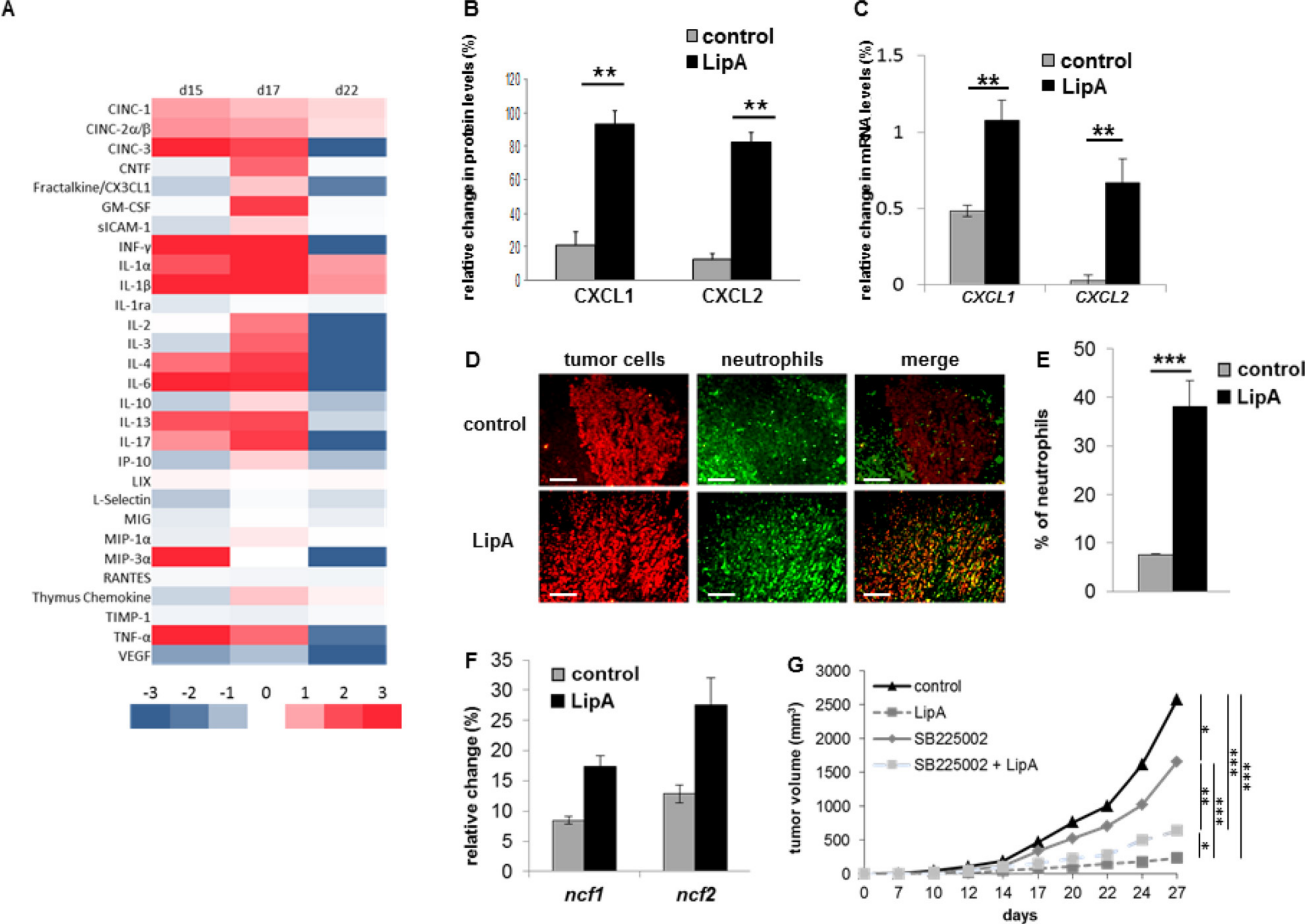
Anti-tumor effect of LipA was correlated with neutrophil infiltration (**A**) The modulation of cytokine and chemokine contents of tumors one day after the first, second and fourth injections (respectively d15, d17 and d22) of LipA or physiological solution was analyzed using antibody arrays and Image J software. (**B**) The relative abundance of CXCL1 and CXCL2 proteins twenty-four hours after the second injection of LipA, (d17) was expressed as a percentage compared to an internal control (100%). (**C**) Six hours after the first injection (d15) of LipA or physiological solution (control), expression levels of cxcl1 and cxcl2 mRNA in tumors were evaluated relative to housekeeping gene gapdh by RT-PCR. (**D**) Tumors were collected at day 17 from LipA treated or control rats. After fixation, 5-µm sections were cut and stained for tumor cells (anti-cytokeratin Ab, red) and neutrophils (anti-HIS48 Ab, green) (scale bars = 50 µm). The yellow staining in the merge panels represents tumor cells very close to neutrophils. Micrographs are representative of at least 3 independent experiments, 4 animals per group. (**E**) The levels of neutrophils present in tumors were determined by counting of these cells in 3 independent slides per animals, 4 animals per group. Shown are the mean % of neutrophils ± SEM. (**F**) At day 17, expression levels of ncf1 and ncf2 mRNA were evaluated relative to housekeeping gene gapdh by RT-PCR. (**G**) CT26-bearing mice were treated with physiological solution (control) or LipA with or without the GZMB inhibitor SB225002. Three days after cell injection, LipA was administrated i.v. every 5 days for 5 times. SB225002 was injected i.p. 24 h before and at the same time than LipA. Results are representative of at least 2 independent experiments with 10 animals per group. (B, C, E–G) Significant difference in Mann–Whitney *U* test, ^*^p < 0.05, ^**^p < 0.01, ^***^p < 0.005.

The LipA treatment induced the regression of tumors and cured 95% of tumor-bearing rats (Figure 1A) that died free of tumor nodules, as previously shown [8]. Typical structures of tumors after the second injection of physiological solution (control) or LipA are shown after hematoxylin-eosin Y (HE) staining (Figure 1B and 1C). Both types of tumors consisted of core of tumor cells surrounded by a distinct ring of non-tumor cells at the edges of nodules. It should be noted that no other tumor sites were observed (data not shown). However, in treated tumors, this ring was thicker than in control tumors (Figure 1C) and the number of tumor cells decreased drastically (Figure 1D). We analyzed the cytokine and chemokine contents of tumors using antibody arrays (Figure 2A). LipA treatment induced lot of modulations in cytokine levels. Furthermore, chemokines belonging to CXCL family such as CXCL1 (CINC-1) and CXCL2 (CINC-2α/β, CINC-3) were significantly higher (approximately five-fold) in treated rats at days 15 and 17 (Figure 2A and 2B). In agreement, tumor *cxcl1* and *cxcl2* mRNA levels were higher after the first injection of LipA compared to control rats (Figure 2C). As a consequence, tumors isolated from treated rats contained many more neutrophils than tumors from control rats (Figure 2D and 2E). In agreement, specific neutrophil mRNA transcripts (*ncf1* and *ncf2*) were higher in treated rats compared to control rats (Figure 2F). However, neutrophils remained at the edges of tumor nodules in control rats whereas they infiltrated the entire nodule after LipA treatment (Figure 2D). Moreover the antitumor effect of LipA in a mouse model of subcutaneous colon cancer was partially decreased by inhibition of neutrophils recruitment using a CXCR2 antagonist, SB225002 (Figure 2G, Supplementary Figure 1A).

### Apoptotic tumor cells are in the vicinity of anti-tumorigenic neutrophils in LipA treated rats

**Figure 3:**
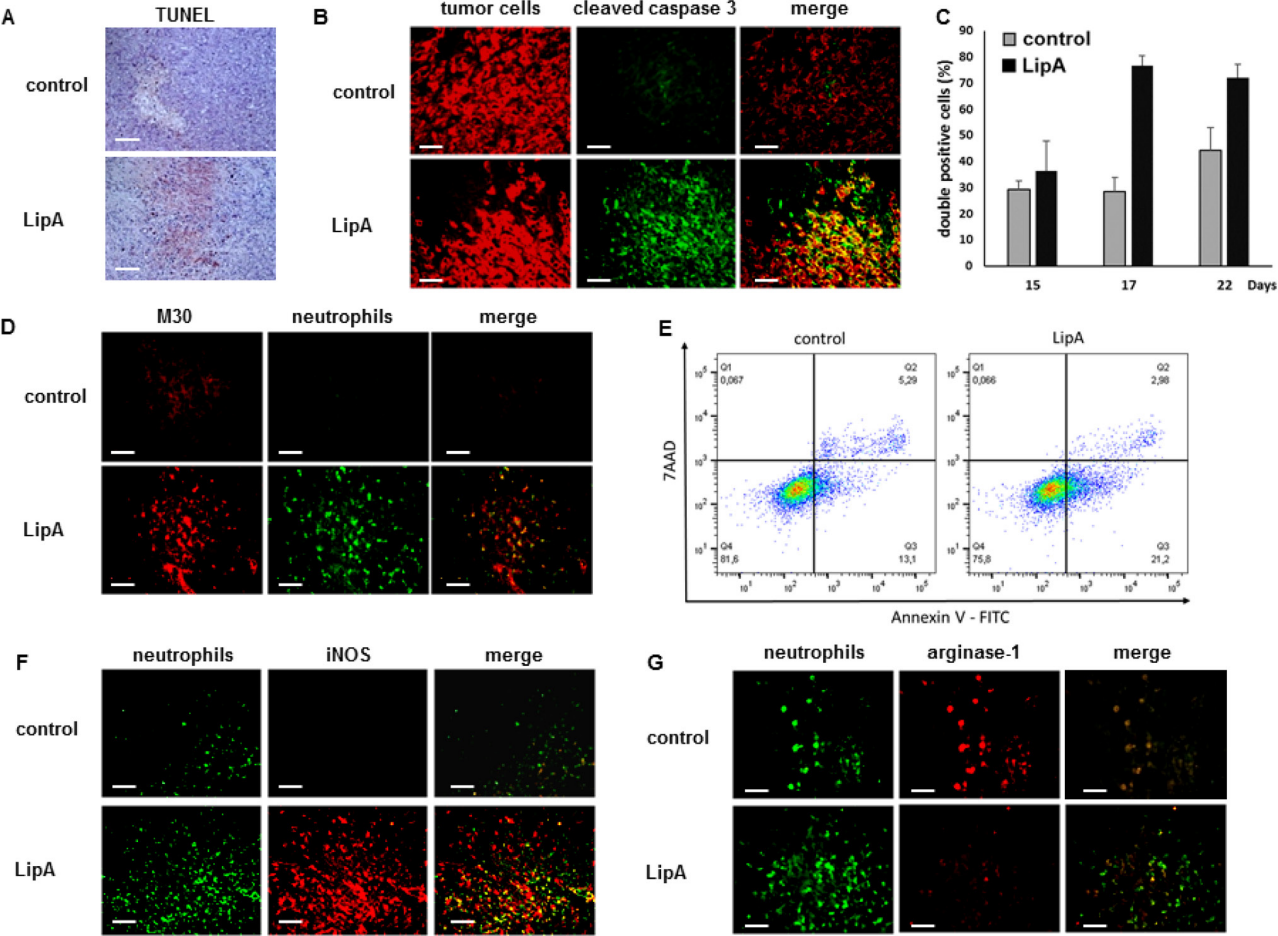
LipA treatment induced tumor cell death in the vicinity of infiltrated anti-tumorigenic neutrophils Tumors from LipA treated or control rats were removed at day 17 (**A**, **B**, **D**, **F**, **G**), fixed and cut into 5-μm cryosections. (A) Apoptotic cells were present in the core of tumors from LipA treated rats but not in tumors from control rats (TUNEL, red). (B) Immunostaining of tumor cells (anti-cytokeratin Ab, red) and cleaved caspase 3 (anti-cleaved caspase 3 Ab, green). (**C**) The levels of tumor cells containing cleaved caspase 3 were determined at days 15, 17 and 22 by counting of these cells in 3 independent slides per animals, 4 animals per group. Shown are the mean % of double positive cells ± SEM. (D) Immunostaining of apoptotic tumor cells (M30 Ab, red) and neutrophils (anti-HIS48 antibody, green). (**E**) The levels of apoptotic tumors cells (AnnV+ cells) was determined using an Annexin V-7AAD staining, after LipA treatment of co-culture of CT26 cells and tumor associated-neutrophils. (F, G) Staining for neutrophils (anti-HIS48 Ab, green) and (F) iNOS (anti-iNOS Ab, red) or (E) arginase-1 (anti-arginase-1 Ab, red). Micrographs are representative of at least 3 independent experiments, 4 animals per group (scale bars = 50 µm).

By TUNEL analysis, we found that apoptotic death occurred in cells that were located in the core of treated tumors (Figure 3A). We confirmed by immunostaining that most apoptotic cells containing cleaved caspase 3 in treated rats were tumor cells (Figure 3B), the percentage of apoptotic tumor cells increasing during treatment (Figure 3C). Moreover, double immunostaining of neutrophils and M30 (a marker of apoptotic epithelial cells) demonstrates that in treated tumors the death of tumor cells occurred near neutrophils (Figure 3D). LipA-induced apoptosis was also detected in the murine CT26 tumor cells co-cultured with tumor-associated neutrophils (Figure 3E), while the LipA treatment in absence of neutrophils did not trigger apoptosis (data not shown). These data revealed the anti-tumor potential of these TANs stimulated by LipA. On the contrary, apoptosis was not detected in control tumors in which neutrophils were at the edges of tumors, distant from tumor cells, and presented a pro-tumorigenic phenotype (N2 state) (Figure 2D and Figure 3F and 3G). However, LipA induced the acquisition of an anti-tumorigenic phenotype by neutrophils (N1 state) with iNOS expression and low arginase-1 content (Figure 3F and 3G).

### The cytotoxic effect of the LipA treatment correlates with the increase of granzyme B (GZMB) into tumor cells

**Figure 4:**
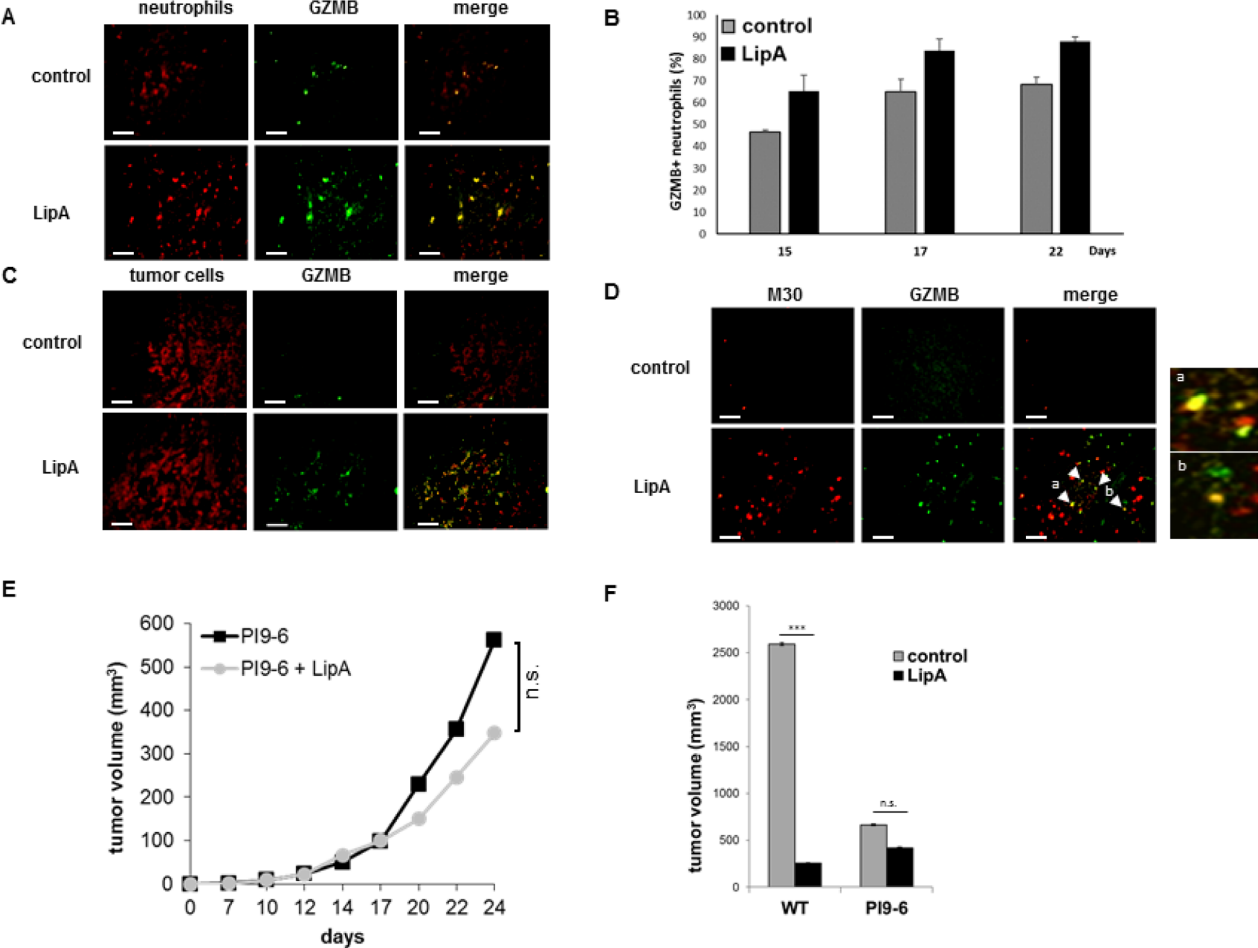
Granzyme B was present in neutrophils and apoptotic tumor cells in LipA treated rats Tumors from rats were removed at day 17, fixed, cut into 5-μm cryosections and stained. (**A**) Tumor associated-neutrophils containing GZMB were double stained (Neutrophils in red, GZMB in green). (**B**) The levels of neutrophils containing GZMB were determined at days 15, 17 and 22 by counting of these cells in 3 independent slides per animals, 4 animals per group. Shown are the mean % of double positive cells ± SEM. (**C**, **D**) Tumor sections were stained for tumor cells (red (C)), or apoptotic tumor cells (anti-M30 Ab, red (D)) and GZMB (anti-GZMB Ab, green (C, D)). Enlargement of regions a and b in figure (D) Micrographs are representative of at least 3 independent experiments, 4 animals per group (scale bars = 50 µm). (**E**, **F**) Three days after s.c. injection of PI9-6 cells (E, F) or CT26 wild type cells (WT, F), mice were treated i.v. with LipA or physiological solution (control) every 5 days for 5 times. Results are representative of at least 2 independent experiments with 10 animals per group. Significant difference in Mann–Whitney *U* test, ^***^p < 0.005, n.s. not significant.

To further characterize the phenotype of tumor-associated neutrophils, we assessed the expression of GZMB, an arm used by NK and cytotoxic CD8 T-lymphocytes cells to kill their targets. Unexpectedly, GZMB was observed only at the edge of tumor sections from control rats, but throughout the whole tumor sections from treated rats (Figure 4A) while the presence of GZMB was associated with a large proportion of neutrophils in both treated and control tumors from rats (Figure 4A) but also in tumor-associated neutrophils from mice (Supplementary Figure 1B). Moreover, GZMB was also found inside tumor cells from treated rats, but not from control rats (Figure 4B). Further, double immunostaining of GZMB and M30, a hallmark of apoptosis, demonstrates that apoptotic tumor cells in treated rats contained GZMB (Figure 4C). The involvement of GZMB in the anti-tumor effect of lipid A has been demonstrated by using cells expressing the serpin B9 (PI9-6 cells), a natural protein inhibitor of GZMB (Figure 4D). In fact, the percentage of tumor growth inhibition induced by LipA is 90% with CT26WT cells but only 37% with CT26 cells expressing serpin B9 (Figure 4E and 4F). Taken together, these data suggest that GZMB from neutrophils contributed to tumor cell apoptosis in LipA treated rats. Some other cells were known to express GZMB like NK cells and T lymphocytes (αβ and γδ). However, these cells were present only at the edges of the tumors, none were detected in the core of tumors from control or treated rats (Supplementary Figure 2). As we showed that tumor cells were located (Figure 1C) and died (Figure 3A, 3B and 3D) in the core of tumor nodules, we deduced that the latter cell types were not involved in tumor cell death in LipA immunotherapy, at least at the beginning of treatment.

**Figure 5:**
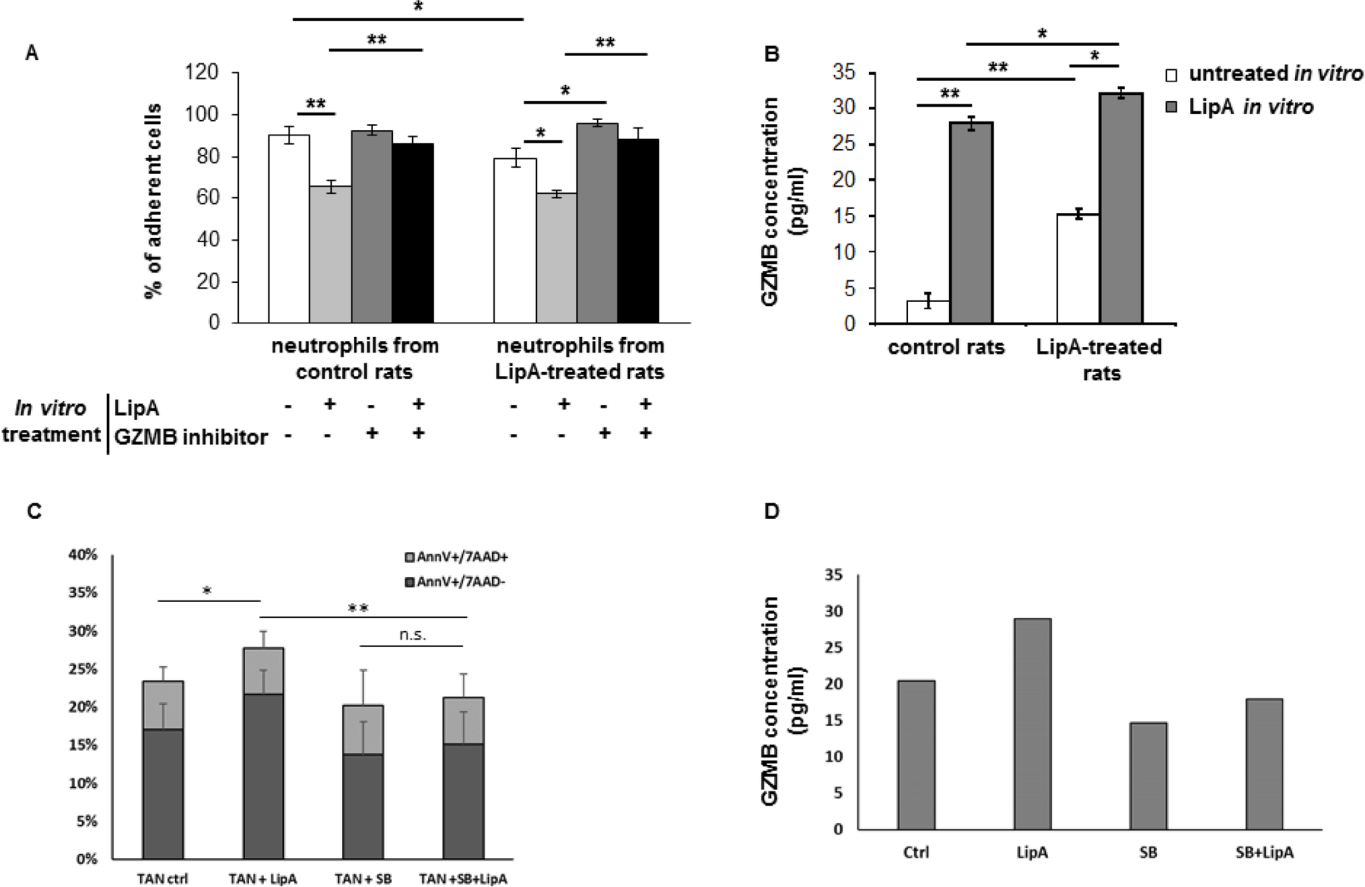
LipA induced neutrophil-mediated cytotoxicity via granzyme B production (**A**, **B**) Spleen neutrophils from LipA treated or control rats were purified and treated *in vitro* with or without 10 µg/ml LipA for 24 h. (A) The toxic effect of conditioned media on tumor target cells cultured in the presence or in the absence of GZMB inhibitor was evaluated using a methylene blue assay. The mean numbers of adherent cells are shown as percentages of the untreated cells (100%). (B) The concentration of GZMB in conditioned media was measured by ELISA. Shown are means ± SEM. (**C**) Neutrophils were incubated or not with LipA (24 h) and stained for granzyme B (green) or DAPI (blue). 3 independent experiments. (C, **D**) Tumor associated-neutrophils were purified from mice, co-cultures with CT26 cells during 24 h in presence or not of LipA and SB203580. (C) The level of apoptotic tumor cells was determined by Annexin V/7AAD staining. (D) The level of GZMB secreted in co-culture of CT26 and TAN treated or not with LipA and SB203580 was determined using an ELISA assay. The results are obtained from one mouse and are representative of those obtained with at least 6 animals. (A–C) Significant difference in Mann–Whitney *U* test, ^*^p < 0.05, ^**^p < 0.01, n.s. not significant.

We also showed that conditioned media of spleen neutrophils from control tumor-bearing rats were not cytotoxic (Figure 5A). However, conditioned media from neutrophils treated with LipA *in vivo* and/or *in vitro* induced cytotoxicity which was GZMB-dependent (Figure 5A). Furthermore, conditioned media from *in vitro* and/or *in vivo* LipA-treated neutrophils contained higher levels of GZMB compared to untreated neutrophils (Figure 5B). Altogether, these results indicated that not only the lipid A induces the release of GZMB of neutrophils but also increases the expression of this enzyme in these cells.

The involvement of GZMB produced by neutrophils in the anti-tumor effect of lipid A has also been demonstrated in a mouse model of colon cancer. Indeed, coculture of TAN induces apoptosis of CT26 tumor cells in response to treatment with LipA, as attested by the increase of Annexin V positive cells (Figure 5C). This cytotoxicity is correlated with the increase of the level of GZMB in co-culture supernatants (Figure 5D). In order to decipher how GZMB was induced in neutrophils treated by LipA, we first tested the effect of some MAPKinases known to modulate GZMB expression. We showed that the p38 MAPKinase inhibitor (SB203580) significantly affected LipA-mediated GZMB increase and cell death (Figure 5C and 5D).

### Tumor microenvironment induces GZMB expression in neutrophils

**Figure 6:**
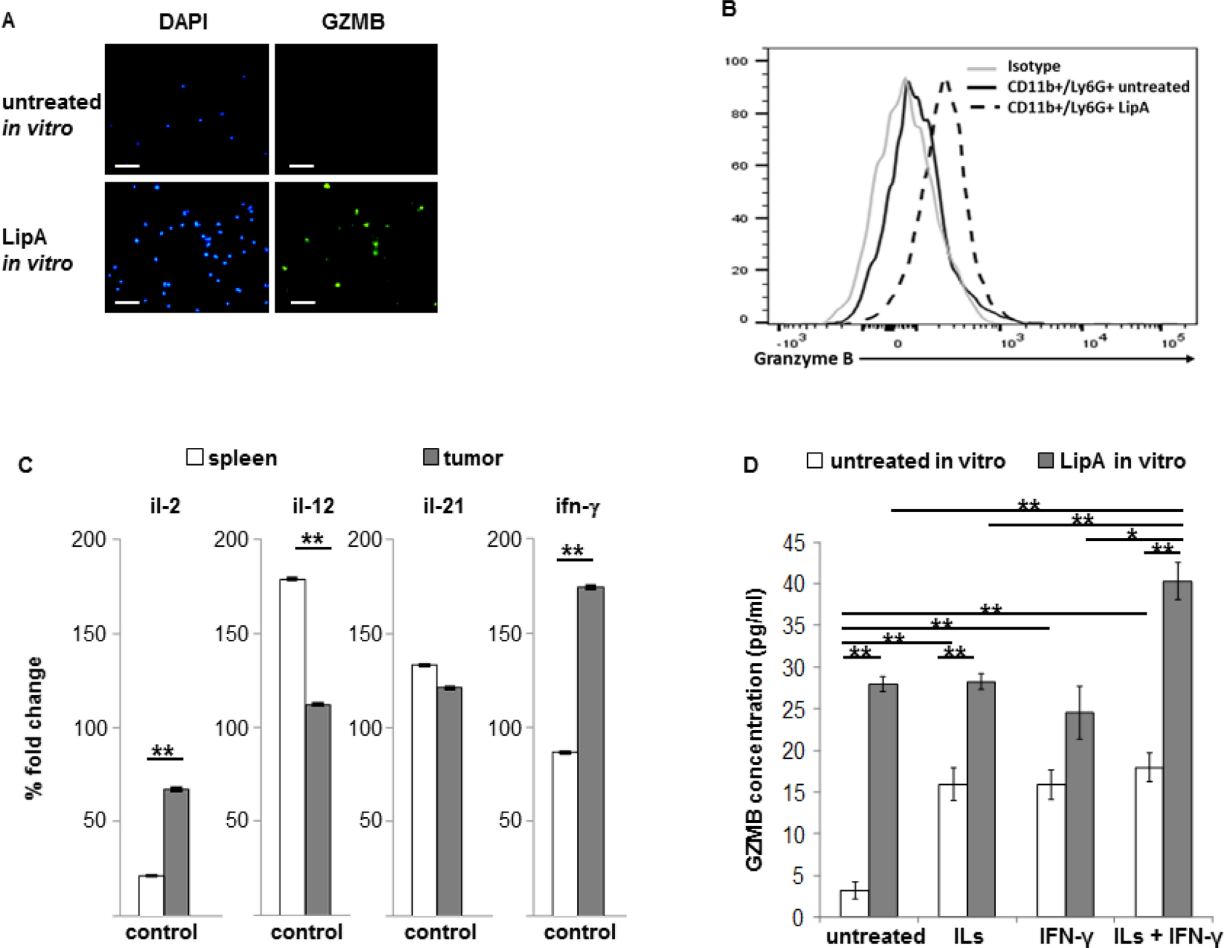
Tumor cytokines and LipA together induced strong expression of granzyme B in spleen and tumor neutrophils (**A**) Spleen neutrophils from rats were purified and incubated or not with LipA (24 h) before staining for granzyme B. The GZMB expression was determined by microscopy. (**B**) Spleen neutrophils from untreated or LipA-treated mice (24 h) were purified before analysis of GZMB by flow cytometry analysis. (A, B) 3 independent experiments. (**C**, **D**) Spleens and tumors from control rats were removed at day 17. (C) Expression levels of *il-2*, *il-12*, *il-21* and *ifn-*γ mRNA were evaluated by RT-PCR and expressed as the mean of percentage of change compared to the level of *gapdh* expression. (D) Neutrophils from spleen were purified as in (A) and incubated for 24 h *in vitro* without or with a cytokine mix (ILs, IL-2 + IL-12 + IL-21) alone, IFN-γ alone or a combination of both (ILs + IFN-γ), with or without of 10 µg/ml LipA for 6 h. Concentration of granzyme B (GZMB) in conditioned media was measured by ELISA. Significant difference in Mann–Whitney *U* test, ^*^p < 0.05, ^**^p < 0.01.

Tumor-associated neutrophils from rats or mice could expressed GZMB, independently of LipA treatment (Figure 4A and Supplementary Figure 1B). However, spleen neutrophils from rats or mice only express GZMB after LipA treatment (Figure 6A and 6B). The tumor microenvironment could therefore stimulate the production of GZMB by TAN. Cytokines like IL-2, IL-12, IL-21 and IFN-γ were known to play a key role in GZMB production in lymphocytes or NK cells [25–27]. Different expression patterns of these cytokines were observed *in vivo* between tumors and spleens in control rats (Figure 6C). IL-21 was expressed at similar levels in tumors and spleens, IL-12 mRNA levels were lower whereas IL-2 and IFN-γ mRNA levels were higher in tumors than in spleens (Figure 6C). Treatment of spleen neutrophils from control rats with an interleukin mix (ILs, IL-2 + IL-12 + IL-21), IFN-γ or ILs plus IFN-γ induced the production of GZMB in these cells (Figure 6D, white bars). This effect was enhanced by the combination of LipA with ILs alone, ILs+IFN-γ but not IFN-γ alone (Figure 6D, grey bars). These results indicate that GZMB expression in neutrophils could be triggered by different cytokines including IFN-γ, IL-2, IL-12 and IL-21 but also by LipA.

### Colon tumor of patients contains granzyme B-expressing neutrophils

Tumor-infiltrating neutrophils were evaluated in a cohort of 35 patients with colon cancer (all patients for which a tumor sample was available had given its written consent). Using hematoxylin-eosin-saffron (HES) staining, we showed that all tumors were infiltrated by neutrophils at variable rates (Table 1). The low number of patients did not allow us to see any significant correlation between the rate of tumor-infiltrating neutrophils and patient’s survival.

**Table 1:** Estimation of neutrophil infiltration into colon tumors

Neutrophil score	Patient proportion (%)
0	0 (0)
1+	14 (40%)
2+	10 (29%)
3+	11 (31%)

Score of tumor infiltration in polymorphonuclear neutrophils (PMN) was determined as: 0; absence of PMN in tumor zones outside ulceration zones, after careful analysis of the entire cut, 1+; presence of PMN isolated or in small clusters of 2–3 elements in the tumor zones outside ulceration zones. These PMN are difficult to find after careful analysis of the entire cut, 2+: presence of PMN isolated or in small clusters in the tumor zones outside ulceration zones. These neutrophils are easy to find after careful analysis of the entire cut and are of interest to more than one area, 3+: Obvious presence of PMN isolated or clustered in several tumor zones outside ulceration zones.

**Figure 7:**
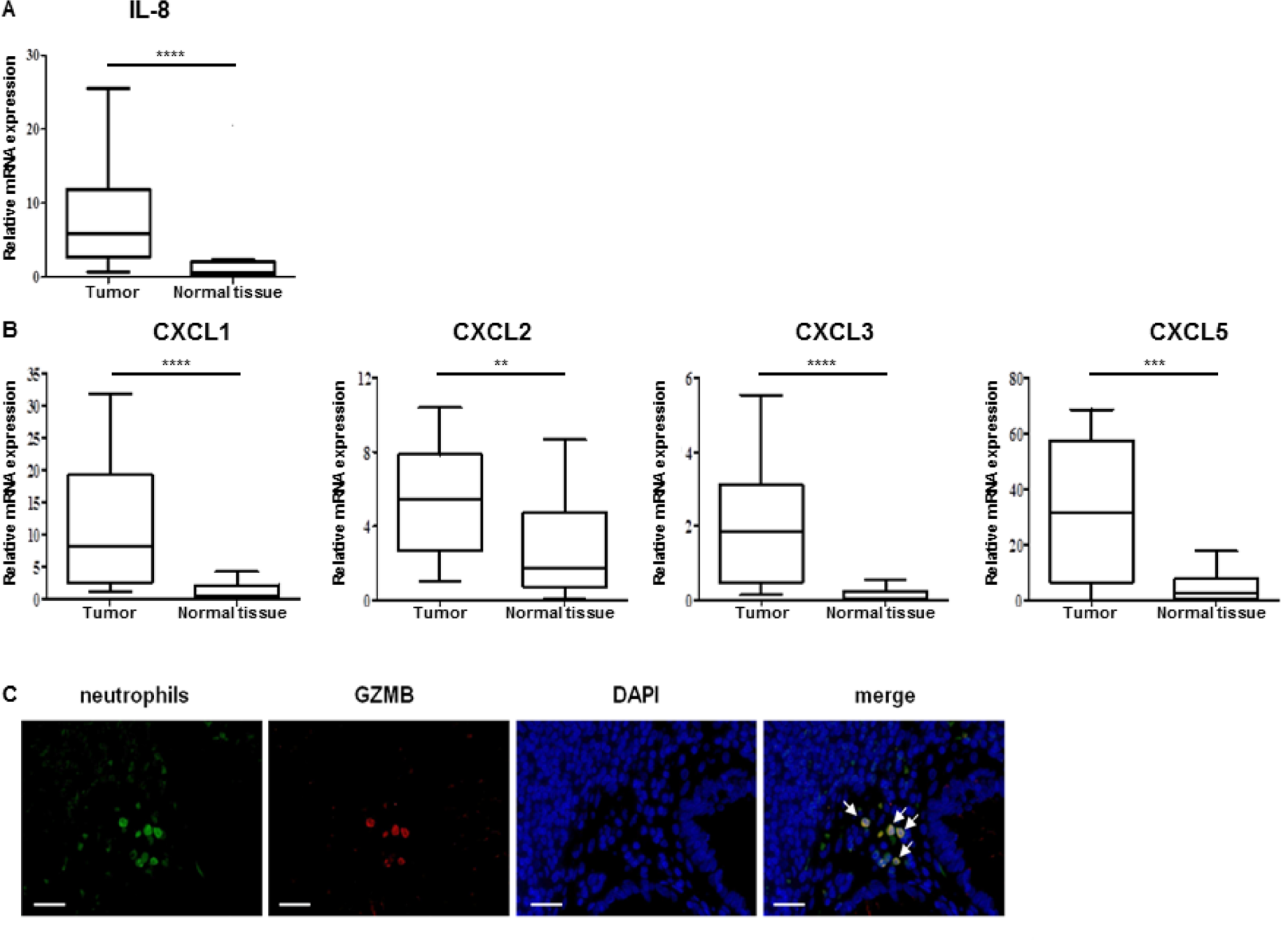
Colon tumor from patients contained neutrophil chemoattractants and granzyme B expressing neutrophils (**A**, **B**) Relative expression level of IL-8 cytokine (A) or CXCL-1, CXCL-2, CXCL-3 and CXCXL-5 mRNA neutrophil chemoattractant (B) into tumor from 25 patients with colon cancer were evaluated by qRT-PCR. β-actin was used as the internal control. Significant difference in Mann–Whitney *U* test, ^*^p < 0.05, ^**^p < 0.01, ^***^p < 0.005, ^****^p < 0.0005. (**C**) Human colon cancer samples were collected and stained for neutrophils (anti-CD66b, green), GZMB (anti-GZMB Ab, red) and for nuclei (DAPI, blue). GZMB was present in neutrophils (yellow signal shown by white arrows in merged panel). Micrographs are representative of at least 20 different samples (scale bar = 50 µm).

We also determined cytokines and chemokines levels in tumor and normal tissues from 25 patients. We showed no significant differences in IL-2, IL-6, and Il-12b mRNA levels between tumor and normal tissues (data not shown). However, the neutrophil CXC chemokine IL-8 mRNA expression was significantly higher (*p* < 0.0001) in tumors as compared to normal tissues (Figure 7A). Furthermore, four other chemokines, CXCL1, 2, 3 and 5, known to attract neutrophils, were strongly expressed in tumors by comparison to the normal tissues (Figure 7B). We also showed that most of tumor-infiltrated neutrophils (CD66b+ cells) expressed GZMB (Figure 7C).

Taken together, these results indicate that animal but also human colon tumors are infiltrate by neutrophils which are characterized by the expression of the GZMB. Such infiltration is correlated with the expression of some chemokines known to attract neutrophils. Stimulation of these cells with a TLR4 agonist triggers the release of GZMB and cancer cell demise, probably at the origin of *in vivo* tumor regression. The involvement of this neutrophil subpopulation in colon tumor outcome needs more investigation.

## DISCUSSION

Here we have identified a subpopulation of colon tumor-infiltrating neutrophils, in rat and mouse models but also in human, which expressed GZMB. When stimulated with a Lipid A analog, this cell type of neutrophils was more recruited into tumors and participated in a GZMB-dependent manner to the antitumor activity of LipA.

Neutrophils are more and more the subject of research on tumor development or tumor therapies. These cells express CXCR1 and CXCR2 at their surface and are recruited by their ligands including CXCL1 and CXCL2 [28] which can be produced by tumor, endothelial cells or fibroblasts [29]. CXCL1 and CXCL2 contribute to mobilizing neutrophils from bone marrow and sustain neutrophil recruitment at sites of inflammation [30–32]. It was conceivable that neutrophils can be attracted to tumors if these later express these ligands. We have demonstrated here that colon tumors from rats showed a rise of CXCL1, CXCL2 after LipA treatment. We also showed that these two chemokines but also CXCL3 and CXCL5 were more expressed in tumors of patients with colon cancer as compared to healthy tissue [33, 34]. The expression of CXCL1 and CXCL2 in human colon tumors has been described [35, 36] and there is a report that CXCL1 is highly expressed in poor invasive tumors [37]. However, description of neutrophils in human colon cancer are limited [38] without significant correlation with prognosis. As far as we know there has been no correlation made between CXCL1, CXCL2 or neutrophils with the effects of therapy on colon cancer in human. It has been shown that CpG oligodeoxynucleotides and a vascular disrupting agent induced the regression of colon tumors in mice, and this correlates with an increase in CXCL1 and neutrophil infiltration in tumors [39, 40]. Furthermore, colon tumor bearing mice receiving cyclophosphamide plus IL-12 gene therapy are more likely to reject tumors when infiltrated by neutrophils [41]. A recent study showed that in early-stage lung cancer, TAN were able to stimulate T-cell response and tumor growth inhibition [42].

One of the most important aspects of this study is that a large proportion of TAN expressed GZMB and was localized into colon tumors from rats, mice and from patients. Further, in lipid A-treated tumors, some neutrophils moved to reside in the vicinity of apoptotic tumor cells. Noteworthy, the apoptotic tumor cells, contiguous to neutrophils, contain GZMB, suggesting that lipid A induces neutrophil GZMB release that contributes to cancer cell demise. In our knowledge, this is the first time that we describe a subpopulation of neutrophil expressing GZMB with an anti-tumor ability. This is all the more important as this type of cells is also present in patients with colon cancer. It would be interesting to know if the prognosis and disease outcome are different in patients in whom tumors are rich or poor in neutrophil expressing GZMB. It has been observed that colorectal cancer infiltration with CD16+ high myeloperoxidase positive myeloid cells correlates with favorable outcome [43, 44]. In another study, tumor-associated neutrophils stimulate T-cell proliferation and interferon gamma releases [42]. Other contradictory results showed that high tumor infiltration by CD66b+, a classical neutrophil marker, cells was associated to severe prognosis in patients with colorectal cancer [45, 46]. A recent study comes to contradict the last ones previously quoted. Indeed, Governa *et al.* reported that CD66b+ cell infiltration in colorectal cancer is associated with increased survival [47].

Our data also showed that GZMB^+^-neutrophils have anti-tumor activity when activated by a TLR4 agonist unlike those devoid of GZMB, and their infiltration into tumor probably contributed to the LipA-mediated tumor cell death. Our conclusions are supported by results showing that the inhibition of GZMB activity in tumor cells affected the anti-tumor activity of LipA. We noted that colon tumor cells expressing the physiologically inhibitor of GZMB, the serpin B9, grew more slowly in mice as compared to wild type cells (80% reduction in tumor volume). This observation underlines the importance of GZMB in the eradication of tumors, at least in our model. The activation of TLR4 of immune cells and their impact on cancer cell death has been well documented. Apetoh *et al*, reported that activation of TLR4 expressed on dendritic cells by the high mobility group box 1 protein (HMGB1) released by dying tumor cells can trigger protective antitumor immunity [48]. Neutrophil activation by BCG, a well-known activator of TLR2 and 4, induced the release of the tumor necrosis factor (TNF)-related apoptosis-inducing ligand (TRAIL) and may play a potential role in initiating antitumor response [22]. A recent report showed that priming of tumor induced macrophages with TLR4 agonist (LPS) alone or in combination with IFN-γ induced a strong anti-tumor immune reaction [49].

In our effort to characterize the signaling pathways involved in the expression of GZMB in neutrophils, we also showed that GZMB was expressed in tumor-infiltrating neutrophils but not in neutrophils from spleen. This could be explaining by the cytokines profile in tumor microenvironment which exhibited differences as compared to spleen. Indeed, GZMB could be induced in immune cells as NK when stimulated with IL-2 and IL-12 in humans, plus IL-21 in mice [25–27]. We showed that some of these cytokines triggered GZMB expression in neutrophils, an effect that was enhanced by the LipA analog. It is well-known that cytokines stimulate host immune response against tumors [50]. The host-derived cytokines exert double function; it can promote proliferation as well as inhibit tumor progression [51]. For instance, the pro-inflammatory cytokines IL-1 and TNF-α promote tumor growth. In contrast, IFN-γ contributes to prevention of carcinogen-induced sarcomas [52] and its loss enhances tumor formation [51]. Although the pro- or anti-tumor response mechanisms of cytokines are not well understood, several reports indicated the expression of certain chemokines in tumor microenvironment promote immunosuppressive or anti-tumor immune cell recruitment. In our study, the increase of CXCL1 and CXCL2 both in rat model and patients with colon cancer correlates with neutrophil recruitment within tumors.

We also found that the LipA analog converts neutrophils from the protumorigenic N2 to the anti-tumor N1 phenotype as attested by the induction of iNOS in LipA-treated neutrophils. This seems to be important for the anti-tumor function of neutrophils mediated by the GZMB. However, the relationship between the expression of iNOS, a hallmark of N1 phenotype, and GZMB expression and release remains to be determined. Furthermore, in search of signaling pathways involved in GZMB expression of neutrophils, we showed that p38 MAPKinase was required for LipA-induced GZMB release from neutrophil and subsequently for neutrophil mediated cytotoxicity.

Overall, we provide strong evidence for the involvement of GZMB produced by neutrophils in immuno-induced tumor regression, widening the field of tumor immunotherapy mechanisms.

## MATERIALS AND METHODS

### Animal tumor models and cell culture

BD-IX rats and Balb/C mice, respectively 2–3 month-old males and 7–8 weeks-old females, were purchased from Charles River Laboratory. Carcinomatosis in BD-IX rats was initiated by i.p. injection of colon cancer cells PROb [53, 54], a cell line obtained from a colon carcinoma induced in BD-IX rat (Martin *et al*, 1983) and maintained at 37° C in HAM’s F10 (Lonza) supplemented with 10% FBS (Lonza). As previously described [8], the treatment began at day 14, when the size of tumor nodules were around 2 to 5 mm in diameter, and consisted of 15 i.v. injections of LipA at 1 mg/kg or of physiological solution (in control rats), at the rate of 3 injections per week. Tumors in Balb/C mice were initiated by s.c. injection of CT26 cells, a mouse colon cancer cell line obtained from the American Type Culture Collection and maintained at 37° C in RPMI (Lonza) supplemented with 10% FBS (Lonza). The treatment began at day 3 and consisted of 5 i.v. injections of LipA at 8 mg/kg or physiological solution (in control mice) every 5 day. The inhibitor of neutrophils (SB225002, Tocris Bioscience) was injected i.p. at 4 mg/kg the day before and at the same time that LipA injections. The studies were reviewed and approved by the Burgundy University ethics committee for animal experimentation. CT26 cells were stably transfected by calcium phosphate method [55] with the pCMV6-Kan:Neo expression plasmid containing the full-length serpin B9 cDNA (OriGene). Clonal populations of CT26 overexpressing serpinB9 were selected by isolation of a single cell into a well of a 96-well plate and were cultured with medium supplemented with neomycin at 1 mg/ml.

### Lipid A analog

OM-174 (OM-Pharma, Meyrin, Switzerland), is a 2-deoxy-6-O-[2-deoxy-2- [(R)-3-dodecanoyloxytetra decanoylamino] -4-O-phospho-β-D-glucopyranosyl] -2- [(R)-3-hydroxytetradecanoylamino] -α-D-glycopyranosyl dihydrogenphosphate.

### Antibodies and reagents

Mouse antibody (mAb) anti-granulocytes (Santa Cruz, clone HIS48) was used to characterize tumor-infiltrating and purified neutrophils. Mouse anti-TCR α/β Ab (AbD Serotec, clone R73), goat polyclonal antibody (pAb) anti-NKp46 Ab (R&D Systems) and mAb anti-TCR γ/δ Ab (AbD Serotec, clone V65) were used to detect T lymphocytes, NK cells and γ/δ T lymphocytes respectively in tumors. Tumor cells were characterized using an anti-cytokeratin mAb (DakoCytomation, clone MNF116). The following Abs were also used: a mouse anti-arginase 1 mAb (clone 19) and a rabbit anti-iNOS pAb (BD Transduction Laboratories), a rabbit anti-cleaved caspase-3 pAb (Cell Signaling), a mouse Anti-Cytokeratin 18 M30 CytoDEATH mAb (Roche, clone M30), a mouse anti CD66b mAb (Biolegend, clone G10F5) and a rabbit anti-granzyme B pAb (Thermo Scientific). Secondary Abs used were anti-mouse or anti-rabbit Alexa Fluor 568 or 488 conjugates from Molecular Probes (Invitrogen). GZMB inhibitor 1 (z-AAD-CMK) was purchased from Merck. Anti-CD11b-Alexa700 mAb (clone M1/70) and Ly6G-Fitc mAb (clone 1A8) (BD Pharmingen), a rat anti-granzyme-B-efluor450 pAb (eBioscience) and anti-CD45-APC/Cy7 mAb (Biolegend, clone 30-F11) were used for cytometry analysis. The following cytokines and inhibitors were added to neutrophil cultures: recombinant rat IL-2 (Biolegend), recombinant mouse IL-21 (eBioscience), IL-12 and IFN-γ (R&D Systems) and p38 MAP Kinase Inhibitor (SB203580, Invivogen).

### Detection of cytokines and chemokines by Ab array

Tumors were harvested, lysed in lysis buffer [56] and an equal amounts of proteins (Dc Protein Assay kit (BioRad) were incubated with rat cytokine array membranes (R&D Systems). The revelation was performed according to the manufacturer’s protocol and optical density was measured with ImageJ software.

### RT-PCR

Total RNA from rat samples was extracted from tumors using the RNeasy kit (Qiagen). RT-PCR were performed with the Qiagen OneStep RT-PCR kit (Qiagen) according to the manufacturer’s protocol. The primer list was in Table 2. The PCR products were analyzed by gel electrophoresis (1.5% agarose) and stained with ethidium bromide (Invitrogen). The amplified cDNA length was evaluated using the 100-bp DNA ladder (Promega). All densitometric analyses were performed with ImageJ software. Total RNA from human samples was extracted from tumors with Trizol (Ambion), qRT-PCR were performed and analyzed with the Sybr Green method (Thermofisher) according to the manufacturer’s instructions. Expression was normalized to the expression of human beta-actin using the 2^e-ΔCT^ method previously described [57].

**Table 2:** Primers used for RT-PCR

Gene	Rat sequence	Human sequence
CXCL-1	GAGAAAGAAGATAGATTGCACCGATG TTCTTCCCGCTCAACACCTTC	GAAAGCTTGCCTCAATCCTG CTTCCTCCTCCCTTCTGGTC
CXCL-2	GTGACACTGAAGAGTTACGATGTCAG CCTGAGGCTCCATAAATGAAAGA	GCTCCTTGCCAGCTCTCCTCCT TGTGGCTATGACTTCGGTTTGGGC
CXCL-3		CGCCCAAACCGAAGTCATAGCCA TGGTAAGGGCAGGGACCACCC
CXCL-5		TGTTGAGAGAGCTGCGTTGCGTT GGAGGCTACCACTTCCACCTTGG
IL-2	AAGCAGGCCACAGAATTGAAAC CAGATGGCTATCCATCTCCTCAG	
IL-8		TGACTTCCAAGCTGGCCGTGG AAACTGCACCTTCACACAGAGCTGC
IL-12	CACTCACATCTGCTGCTCCAC CTGGCACGCCACTGAGTACTTC	
IL-21	CAGCTCCACAAGATGTAAAGGGGC CCATGTGCCTCTGTTTATTTCCTGTCC	
IFN-γ	CTCTTGGATATCTGGAGGAACTGG CGACTCCTTTTCCGCTTCCT	
Ncf1	CACCGAGATCTACGAGTTCC TCCCATGAGGCTGTTGAAGTAC	
Ncf2	GAAAGCATGAAGGATGCCTGG ATAGCACCAAGATCACATCTCCTTCC	
GAPDH	GGCACAGTCAAGGCTGAGAATG ATGGTGGTGAAGACGCCAGTA	

### ELISA

Granzyme B concentrations in spleen neutrophil supernatants from rats or tumor associated-neutrophils from mice were detected by ELISA (Uscn Life Science Inc and R&D Systems) according to the manufacturer’s protocol. Absorbance was read on a spectrophotometer (Asys UVM 340) at 450 nm wavelength (wavelength correction at 540 nm for mice ELISA).

### Histopathology

Paraffin-embedded samples were cut into 5 µm thick. Sections were deparaffined, rehydrated and stained with hematoxylin-eosinY (HE) or May-Grünwald Giemsa (MGG) for rat samples and Hematoxylin-eosin-saffron (HES) for human samples. Apoptotic cells were detected by TUNEL kit (Millipore) according to the manufacturer’s protocol.

### Immunohistofluorescence (IHF) and immunofluorescence (IF)

For IHF, fixed tumors were embedded in *OCT* (Labonord) after impregnation with 20% *sucrose*/PBS. Sections were cut into 5 µm thick and fixed for 10 min in cold acetone. Antigen retrieval was performed (citrate buffer pH 7.3, 96° C, 20 min) before the blocking of nonspecific sites with 3% BSA. For IF, neutrophils (1 × 10^4^) were plated in Lab-Tek II (Nalge Nunc), fixed in 4% paraformaldehyde, permeabilized in 0.2% Triton X-100 and incubated with 3% BSA. Slides for IHF and IF were incubated with primary antibodies in 1% BSA/PBS/0.1% Tween, then with appropriate secondary antibodies in 1% BSA/PBS/0.1% Tween. Slides were mounted using Prolong Gold (Invitrogen). Images from rat colon cancer were captured on a Nikon Eclipse E400 epifluorescent microscope at 40x magnification, images from human colon cancer were captured on a Axio Imager 2 microscope with Axiovision software.

### Apoptotic cell detection *in vitro*

Apoptotic cells was determined by Annexin V/7 AAD kit (BD Biosciences) according to the manufacturer’s protocol.

### Neutrophil isolation and culture

Neutrophils from rats were purified from spleens removing of red blood cells by osmotic lysis and purification with Ab-anti HIS48 coupled to magnetic beads (Miltenyi Biotec). Neutrophils (98% purity) were cultivated in Ham’s F-10 medium supplemented with 10% heat-inactivated FBS and 1% penicillin/streptomycin/amphotericin B (Lonza). Neutrophils were pretreated for 18 h with 1 ng/mL recombinant IL-2, 1 ng/mL IL-12, 100 ng/mL IL-21 and 250 U/mL IFN-γ before treatment for 6 h with or without 10 µg/mL OM-174. Neutrophils from mice were purified from tumors after mechanic and enzymatic dissociation in solution of RPMI (Dutscher), FBS 2% (Dutscher), Collagenase 1 mg/mL (Sigma-aldrich), DNase 0.1mg/mL (Sigma-aldrich), Hepes 25 mM (Gibco) for one hour at 37° C. After removing red blood cells by osmotic lysis and purification with anti-Ly-6G Microbead Kit (Miltenyi), TAN were co-cultivated with CT26 cells (5:1 ratio) in RPMI medium supplemented with 10 FBS and 1% penicillin/streptomycin/amphotericin B (Dutscher) and treated (18 h) or not with LipA at 500 ng/mL and/or SB203580 at 2,5 µM.

### Methylene blue toxicity assays

After 24 h the supernatants from the above neutrophil cultures were collected and added to 2 × 10^4^ PROb cells that were then treated with or without 50 µM GZMB inhibitor-1 in 96-well microplates for 24 h. The cytotoxicity of neutrophil conditioned media to PROb cells was evaluated using the methylene blue assay as previously described [7].

### Quantification of human tumor-infiltrated neutrophils

The quantification of this infiltration was carried out with the help of a pathologist from slides colored in a standard manner by the HES. The tumors were then classified into 4 groups according to the criteria defined in the Table 1 legend. The same analysis was then redone from the slides colored in MGG.

### Granzyme-B detection by flow cytometry

Spleen neutrophils from mice were incubated for 4 h at 37° C in RPMI medium supplemented with 10% FBS and Golgi Stop (BD Biosciences). Nonspecific sites were saturated in the FCSB solution (eBiosciences) and then incubated with the membrane antibodies (CD11b, CD45, Ly-6G) in the FCSB solution. After fixation and permeabilization (Fixation/Permeabilization solution, BD Biosciences), neutrophils are incubated with an anti-Granzyme-B. The cells were analyzed by flow cytometry on BD FACSCanto™ 10.

### Statistical analyses

Experimental data are expressed as means ± SEM. Statistical analysis was performed using StatView software. The Mann–Whitney *U* test was used to compare data between two treatment groups. Differences were considered statistically significant as follows: ^*^P ≤ 0.05; ^**^P ≤ 0.01; ^***^P ≤ 0.005, and ^****^P ≤ 0.0005.

## SUPPLEMENTARY MATERIALS FIGURES



## References

[1] Sylvester RJ, van der MEIJDEN AP, Lamm DL (2002). Intravesical bacillus Calmette-Guerin reduces the risk of progression in patients with superficial bladder cancer: a meta-analysis of the published results of randomized clinical trials. J Urol.

[2] Heldwein KA, Liang MD, Andresen TK, Thomas KE, Marty AM, Cuesta N, Vogel SN, Fenton MJ (2003). TLR2 and TLR4 serve distinct roles in the host immune response against Mycobacterium bovis BCG. J Leukoc Biol.

[3] Wang RF, Miyahara Y, Wang HY (2008). Toll-like receptors and immune regulation: implications for cancer therapy. Oncogene.

[4] Takeuchi O, Hoshino K, Kawai T, Sanjo H, Takada H, Ogawa T, Takeda K, Akira S (1999). Differential roles of TLR2 and TLR4 in recognition of gram-negative and gram-positive bacterial cell wall components. Immunity.

[5] Parr I, Wheeler E, Alexander P (1973). Similarities of the anti-tumour actions of endotoxin, lipid A and double-stranded RNA. Br J Cancer.

[6] Isambert N, Fumoleau P, Paul C, Ferrand C, Zanetta S, Bauer J, Ragot K, Lizard G, Jeannin JF, Bardou M (2013). Phase I study of OM-174, a lipid A analogue, with assessment of immunological response, in patients with refractory solid tumors. BMC Cancer.

[7] Gautier T, Paul C, Deckert V, Desrumaux C, Klein A, Labbé J, Le Guern N, Athias A, Monier S, Hammann A, Bettaieb A, Jeannin JF, Lagrost L (2010). Innate immune response triggered by triacyl lipid A is dependent on phospholipid transfer protein (PLTP) gene expression. FASEB J.

[8] Onier N, Hilpert S, Arnould L, Saint-Giorgio V, Davies JG, Jeannin JF (1999). Cure of colon cancer metastasis in rats with the new lipid A OM 174. Apoptosis of tumor cells and immunization of rats. Clin Exp Metastasis.

[9] Onier N, Hilpert S, Reveneau S, Arnould L, Saint-Giorgio V, Exbrayat JM, Jeannin JF (1999). Expression of inducible nitric oxide synthase in tumors in relation with their regression induced by lipid A in rats. Int J Cancer.

[10] Seignez C, Martin A, Rollet CE, Racoeur C, Scagliarini A, Jeannin JF, Bettaieb A, Paul C (2014). Senescence of tumor cells induced by oxaliplatin increases the efficiency of a lipid A immunotherapy via the recruitment of neutrophils. Oncotarget.

[11] Lamrani M, Sassi N, Paul C, Yousfi N, Boucher JL, Gauthier N, Labbé J, Seignez C, Racoeur C, Athias A, Guerreiro R, Vergely C, Rochette L (2015). TLR4/IFNγ pathways induce tumor regression via NOS II-dependent NO and ROS production in murine breast cancer models. OncoImmunology.

[12] Afonina IS, Cullen SP, Martin SJ (2010). Cytotoxic and non-cytotoxic roles of the CTL/NK protease granzyme B.. Immunol Rev.

[13] Cullen SP, Martin SJ (2008). Mechanisms of granule-dependent killing. Cell Death Differ.

[14] Hochegger K, Eller P, Rosenkranz AR (2004). Granzyme A: an additional weapon of human polymorphonuclear neutrophils (PMNs) in innate immunity?. Blood.

[15] Wagner C, Iking-Konert C, Denefleh B, Stegmaier S, Hug F, Hänsch GM (2004). Granzyme B and perforin: constitutive expression in human polymorphonuclear neutrophils. Blood.

[16] Wagner C, Stegmaier S, Hänsch GM (2008). Expression of granzyme B in peripheral blood polymorphonuclear neutrophils (PMN), myeloid cell lines and in PMN derived from haemotopoietic stem cells *in vitro*. Mol Immunol.

[17] Grossman WJ, Ley TJ (2004). Granzymes A and B are not expressed in human neutrophils. Blood.

[18] Metkar SS, Froelich CJ (2004). Human neutrophils lack granzyme A, granzyme B, and perforin. Blood.

[19] Martin P, Wallich R, Pardo J, Müllbacher A, Munder M, Modolell M, Simon MM (2005). Quiescent and activated mouse granulocytes do not express granzyme A and B or perforin: similarities or differences with human polymorphonuclear leukocytes?. Blood.

[20] Nozawa H, Chiu C, Hanahan D (2006). Infiltrating neutrophils mediate the initial angiogenic switch in a mouse model of multistage carcinogenesis. Proc Natl Acad Sci U S A.

[21] Fridlender ZG, Sun J, Kim S, Kapoor V, Cheng G, Ling L, Worthen GS, Albelda SM (2009). Polarization of tumor-associated neutrophil phenotype by TGF-beta: “N1” versus “N2” TAN. Cancer Cell.

[22] Kemp TJ, Ludwig AT, Earel JK, Moore JM, Vanoosten RL, Moses B, Leidal K, Nauseef WM, Griffith TS (2005). Neutrophil stimulation with Mycobacterium bovis bacillus Calmette-Guerin (BCG) results in the release of functional soluble TRAIL/Apo-2L. Blood.

[23] Rosevear HM, Lightfoot AJ, O'Donnell MA, Griffith TS (2009). The role of neutrophils and TNF-related apoptosis-inducing ligand (TRAIL) in bacillus Calmette-Guérin (BCG) immunotherapy for urothelial carcinoma of the bladder. Cancer Metastasis Rev.

[24] Suttmann H, Riemensberger J, Bentien G, Schmaltz D, Stöckle M, Jocham D, Böhle A, Brandau S (2006). Neutrophil granulocytes are required for effective Bacillus Calmette-Guérin immunotherapy of bladder cancer and orchestrate local immune responses. Cancer Res.

[25] Brady J, Hayakawa Y, Smyth MJ, Nutt SL (2004). IL-21 induces the functional maturation of murine NK cells. J Immunol.

[26] Fehniger TA, Cai SF, Cao X, Bredemeyer AJ, Presti RM, French AR, Ley TJ (2007). Acquisition of murine NK cell cytotoxicity requires the translation of a pre-existing pool of granzyme B and perforin mRNAs. Immunity.

[27] Zhang B, Zhang J, Tian Z (2008). Comparison in the effects of IL-2, IL-12, IL-15 and IFNalpha on gene regulation of granzymes of human NK cell line NK-92. Int Immunopharmacol.

[28] Mantovani A, Savino B, Locati M, Zammataro L, Allavena P, Bonecchi R (2010). The chemokine system in cancer biology and therapy. Cytokine Growth Factor Rev.

[29] Lazennec G, Richmond A (2010). Chemokines and chemokine receptors: new insights into cancer-related inflammation. Trends Mol Med.

[30] Armstrong DA, Major JA, Chudyk A, Hamilton TA (2004). Neutrophil chemoattractant genes KC and MIP-2 are expressed in different cell populations at sites of surgical injury. J Leukoc Biol.

[31] Rittner HL, Mousa SA, Labuz D, Beschmann K, Schäfer M, Stein C, Brack A (2006). Selective local PMN recruitment by CXCL1 or CXCL2/3 injection does not cause inflammatory pain. J Leukoc Biol.

[32] Wengner AM, Pitchford SC, Furze RC, Rankin SM (2008). The coordinated action of G-CSF and ELR + CXC chemokines in neutrophil mobilization during acute inflammation. Blood.

[33] Jamieson T, Clarke M, Steele CW, Samuel MS, Neumann J, Jung A, Huels D, Olson MF, Das S, Nibbs RJ, Sansom OJ (2012). Inhibition of CXCR2 profoundly suppresses inflammation-driven and spontaneous tumorigenesis. J Clin Invest.

[34] Coffelt SB, Wellenstein MD, de Visser KE (2016). Neutrophils in cancer: neutral no more. Nat Rev Cancer.

[35] McLean MH, Murray GI, Stewart KN, Norrie G, Mayer C, Hold GL, Thomson J, Fyfe N, Hope M, Mowat NA, Drew JE, El-Omar EM (2011). The inflammatory microenvironment in colorectal neoplasia. PLoS One.

[36] Wen Y, Giardina SF, Hamming D, Greenman J, Zachariah E, Bacolod MD, Liu H, Shia J, Amenta PS, Barany F, Paty P, Gerald W, Notterman D (2006). GROalpha is highly expressed in adenocarcinoma of the colon and down-regulates fibulin-1. Clin Cancer Res.

[37] Chiu ST, Hsieh FJ, Chen SW, Chen CL, Shu HF, Li H (2005). Clinicopathologic correlation of up-regulated genes identified using cDNA microarray and real-time reverse transcription-PCR in human colorectal cancer. Cancer Epidemiol Biomarkers Prev.

[38] Roxburgh CS, Wallace AM, Guthrie GK, Horgan PG, McMillan DC (2010). Comparison of the prognostic value of tumour- and patient-related factors in patients undergoing potentially curative surgery for colon cancer. Colorectal Dis.

[39] Sharma S, Karakousis CP, Takita H, Shin K, Brooks SP (2004). Cytokines and chemokines are expressed at different levels in small and large murine colon-26 tumors following intratumoral injections of CpG ODN. Neoplasia.

[40] Wang LC, Thomsen L, Sutherland R, Reddy CB, Tijono SM, Chen CJ, Angel CE, Dunbar PR, Ching LM (2009). Neutrophil influx and chemokine production during the early phases of the antitumor response to the vascular disrupting agent DMXAA (ASA404). Neoplasia.

[41] Medina-Echeverz J, Fioravanti J, Zabala M, Ardaiz N, Prieto J, Berraondo P (2011). Successful colon cancer eradication after chemoimmunotherapy is associated with profound phenotypic change of intratumoral myeloid cells. J Immunol.

[42] Eruslanov EB, Bhojnagarwala PS, Quatromoni JG, Stephen TL, Ranganathan A, Deshpande C, Akimova T, Vachani A, Litzky L, Hancock WW, Conejo-Garcia JR, Feldman M, Albelda SM, Singhal S (2014). Tumor-associated neutrophils stimulate T cell responses in early-stage human lung cancer. J Clin Invest.

[43] Sconocchia G, Zlobec I, Lugli A, Calabrese D, Iezzi G, Karamitopoulou E, Patsouris ES, Peros G, Horcic M, Tornillo L, Zuber M, Droeser R, Muraro MG (2011). Tumor infiltration by FcγRIII (CD16)+ myeloid cells is associated with improved survival in patients with colorectal carcinoma. Int J Cancer.

[44] Droeser RA, Hirt C, Eppenberger-Castori S, Zlobec I, Viehl CT, Frey DM, Nebiker CA, Rosso R, Zuber M, Amicarella F, Iezzi G, Sconocchia G, Heberer M (2013). High myeloperoxidase positive cell infiltration in colorectal cancer is an independent favorable prognostic factor. PLoS One.

[45] Rao HL, Chen JW, Li M, Xiao YB, Fu J, Zeng YX, Cai MY, Xie D (2012). Increased intratumoral neutrophil in colorectal carcinomas correlates closely with malignant phenotype and predicts patients' adverse prognosis. PLoS One.

[46] Yamamoto T, Kawada K, Itatani Y, Inamoto S, Okamura R, Iwamoto M, Miyamoto E, Chen-Yoshikawa TF, Hirai H, Hasegawa S, Date H, Taketo MM, Sakai Y (2017). Loss of SMAD4 Promotes Lung Metastasis of Colorectal Cancer by Accumulation of CCR1+ Tumor-Associated Neutrophils through CCL15-CCR1 Axis. Clin Cancer Res.

[47] Governa V, Trella E, Mele V, Tornillo L, Amicarella F, Cremonesi E, Muraro MG, Xu H, Droeser R, Däster SR, Bolli M, Rosso R, Oertli D (2017). The Interplay Between Neutrophils and CD8(+) T Cells Improves Survival in Human Colorectal Cancer. Clin Cancer Res.

[48] Apetoh L, Ghiringhelli F, Tesniere A, Criollo A, Ortiz C, Lidereau R, Mariette C, Chaput N, Mira JP, Delaloge S, André F, Tursz T, Kroemer G, Zitvogel L (2007). The interaction between HMGB1 and TLR4 dictates the outcome of anticancer chemotherapy and radiotherapy. Immunol Rev.

[49] Prakash H, Nadella V, Singh S, Schmitz-Winnenthal H (2016). CD14/TLR4 priming potentially recalibrates and exerts anti-tumor efficacy in tumor associated macrophages in a mouse model of pancreatic carcinoma. Sci Rep.

[50] Lee S, Margolin K (2011). Cytokines in cancer immunotherapy. Cancers (Basel).

[51] Dranoff G (2004). Cytokines in cancer pathogenesis and cancer therapy. Nat Rev Cancer.

[52] Shankaran V, Ikeda H, Bruce AT, White JM, Swanson PE, Old LJ, Schreiber RD (2001). IFNgamma and lymphocytes prevent primary tumour development and shape tumour immunogenicity. Nature.

[53] Jeannin JF, Onier N, Lagadec P, von Jeney N, Stütz P, Liehl E (1991). Antitumor effect of synthetic derivatives of lipid A in an experimental model of colon cancer in the rat. Gastroenterology.

[54] Martin F, Caignard A, Jeannin JF, Leclerc A, Martin M (1983). Selection by trypsin of two sublines of rat colon cancer cells forming progressive or regressive tumors. Int J Cancer.

[55] Kingston RE, Chen CA, Okayama H (2001). Calcium phosphate transfection. Curr Protoc Immunol.

[56] Leon-Bollotte L, Subramaniam S, Cauvard O, Plenchette-Colas S, Paul C, Godard C, Martinez-Ruiz A, Legembre P, Jeannin JF, Bettaieb A (2011). S-nitrosylation of the death receptor fas promotes fas ligand-mediated apoptosis in cancer cells. Gastroenterology.

[57] Livak KJ, Schmittgen TD (2001). Analysis of relative gene expression data using real-time quantitative PCR and the 2(-Delta Delta C(T)) Method. Methods.

