# Supplementary material for: Tumor-derived granzyme B-expressing neutrophils acquire antitumor potential after lipid A treatment
Source: Oncotarget. 2018 Jun 19;9(47):28364–78. doi: 10.18632/oncotarget.25342 (PMC6033356; doi:10.18632/oncotarget.25342)
Supplement: Supplementary file 1 [file oncotarget-09-28364-s001.pdf]

Tumor-derived granzyme B-expressing neutrophils acquire antitumor potential after lipid A treatment

SUPPLEMENTARY MATERIALS

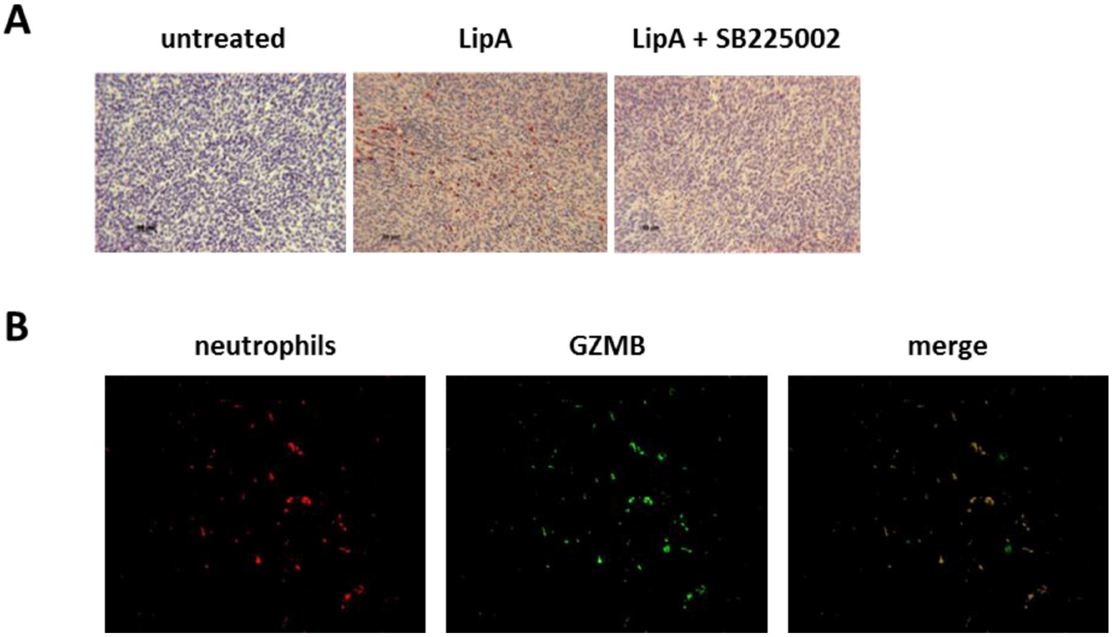

Supplementary Figure 1: Infiltration of GZMB-expressing neutrophils in tumor-bearing mice. Tumors from mice were treated with physiological solution (control) or LipA with or without SB225002. (**A**) Tumors were collected at day 24 and fixed. Five-um cryosections were stained for neutrophils (anti-HIS48 Ab, brown). Results are representative of at least 2 independent experiments with 10 animals per group. (**B**) Expression of GZMB in tumor associated-neutrophils was determined by stained for neutrophils (anti-Ly6G, red) and GZMB (anti-GZMB Ab, green). Micrographs are representative of at least 3 independent experiments, 10 animals per group.

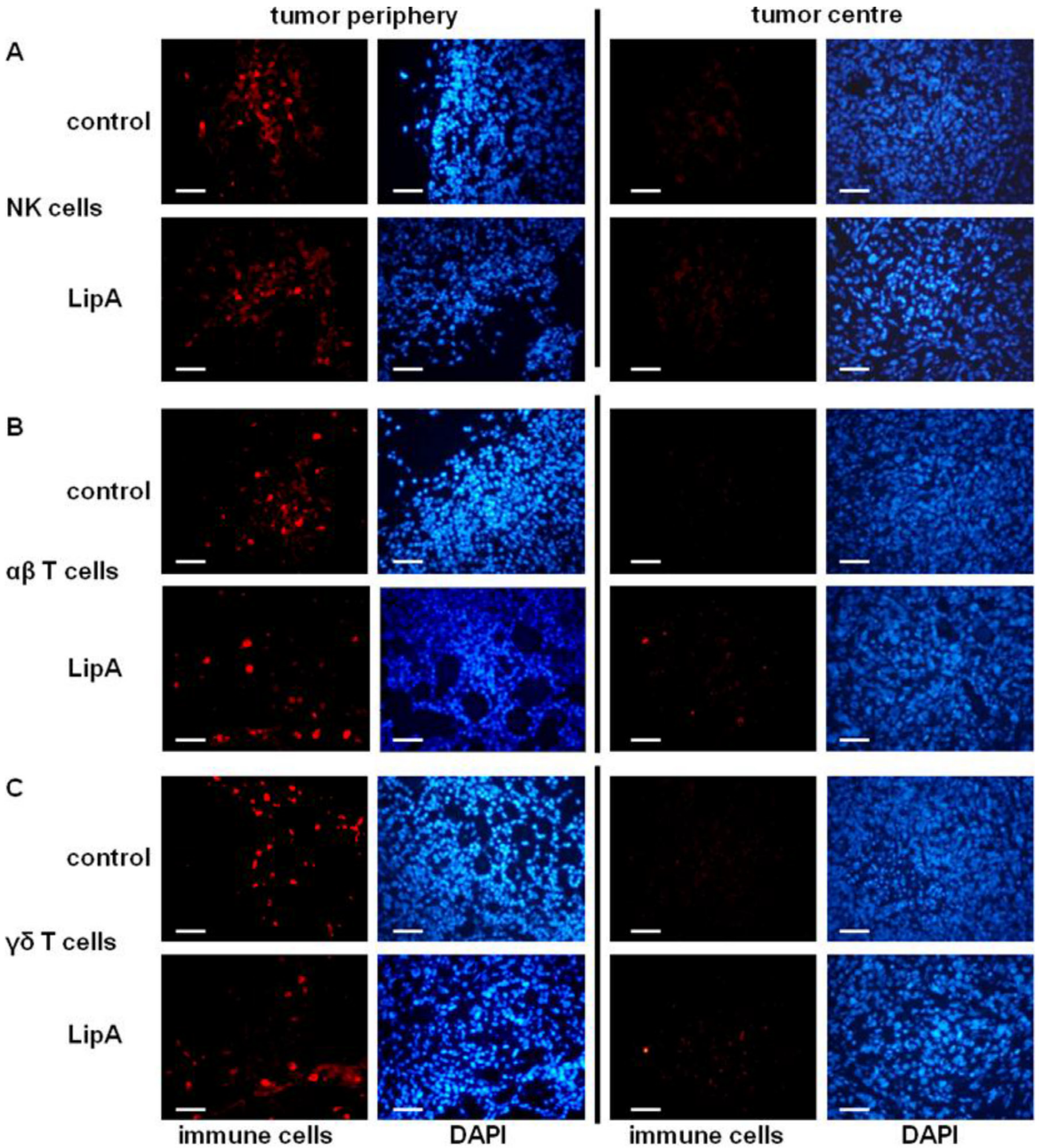

Supplementary Figure 2: NK cells, αβT or γδT lymphocytes remained at the periphery of tumors in LipA treated rats. Tumors from LipA treated or control rats were removed at day 17 and then 5-mm cryosections were prepared and stained (red) for (**A**) NK cells (anti-NKp46 Ab), (**B**) α/β T lymphocytes (anti-TCRα/β Ab) or (**C**) γ/δ T lymphocytes (anti-TCRγ/δ Ab), and for nuclei (DAPI, blue). Left panels show tumor edges, right panels show tumor cores. Micrographs are representative of at least 3 independent experiments, 4 animals per group (scale bars = 50 µm).
